# Persistent Organic Pollutants in Fresh Water Ecosystems

**DOI:** 10.1155/2013/303815

**Published:** 2013-03-06

**Authors:** Fu-Liu Xu, Sven Erik Jorgensen, Yoshihisa Shimizu, Eugen Silow

**Affiliations:** ^1^College of Urban and Environmental Sciences, Peking University, Beijing 100871, China; ^2^Department of Environmental Chemistry, Institute of Analytical and Pharmaceutical Chemistry, Copenhagen University, 2100 Copenhagen Ø, Denmark; ^3^Research Center for Environmental Quality Control, Kyoto University, Otsu, Kyoto 520-0811, Japan; ^4^Institute of Biology, Irkutsk State University, Irkutsk 664003, Russia

Persistent organic pollutants (POPs) are globally concerned pollutants due to their widespread occurrence, long-term persistence, strong resistance, long-range transportation, high bioaccumulation, and potentially significant impacts on human health and ecosystems. Some legacy POPs, such as hexachlorocyclohexanes (HCHs), dichlorodiphenyltrichloroethane (DDT), and polychlorinated biphenyls (PCBs), are still frequently detectable in the environment, although they have been banned or restricted for decades. For some emerging POPs, such as Polybrominated Diphenyl Ethers (PBDEs), Perfluorooctane Sulfonate (PFOS), and Polycyclic Aromatic Hydrocarbons (PAHs), their concentrations in the environment would be increased with social and economic development. Freshwater ecosystems play a pivotal role in supplying drinking water, fisheries, and recreation and in maintaining regional ecological balance and sustainable socioeconomic development, but the world's freshwater ecosystems are generally suffering from POPs pollution. Therefore, it is very meaningful to understand the environmental behaviors, processes, effects, and risks of POPs in freshwater ecosystems. This special issue would provide a window to show some study efforts in such fields. 

9 papers selected form 17 submitted ones are published in the Special Issue on Persistent Organic Pollutants (POPs) in Fresh Water Ecosystems (POPFWEs). The studied contaminants covered legacy and emerging POPs including PAHs, organochlorine pesticides (OCPs) (especially HCHs and DDTs), and PBDEs. The studied media included the water, sediments, fishes, air, and soil. The contents covered the distributions in multimedia, source apportionment, transfer and transformation process, ecological and health risk assessment, and fate modeling. Except for one study in upland Irish headwater lake catchments, other studies were related to the Chinese lakes including Lake Chao, Lake Baiyangdian, Chinese reservoir (Guanting Reservoir), and the Chinese Tianjin coastal area. The air backward trajectories model, dynamic fugacity model, and species sensitivity distribution (SSD) model were developed and applied to the potential secondary source analysis, multi-media modeling, and ecological risk assessment, respectively. 

Although some OCPs such as DDT, lindane, chlordane, mirex, aldrin, dieldrin, and endrin have been banned and their residual levels have gradually decreased since the 1980s, these OCPs could still be detected in various environmental and biological media. In this special issue, the OCPs in Lake Chaohu, the fifth largest lake and one of the most polluted lakes in China, were well studied. The residues, distributions, sources, and ecological risks of OCPs in the water and the simulation of the fate and seasonal variations of *α*-hexachlorocyclohexane (*α*-HCH) in Lake Chaohu were studied in “*Residues, distributions, sources, and ecological risks of OCPs in the water from Lake Chaohu, China*” and *“Simulation of the fate and seasonal variations of *α*-hexachlorocyclohexane in Lake Chaohu using a dynamic fugacity model*,*”* respectively. The levels, temporal-spatial variations, and sources of OCPs in ambient air of Lake Chaohu were investigated in *“Levels, temporal-spatial variations, and sources of organochlorine pesticides in ambient air of Lake Chaohu, China*,*”* since the air would serve as a very important source of OCPs in the water. 

In China, the PAH emissions have been increasing greatly due to the increasing energy demand associated with rapid population growth and economic development and to the low efficiency of energy utilization. The threaten of PAHs pollution to ecosystem and human health has become more and more serious in China. In this special issue, there are three articles dealing with the PAHs in the aquatic ecosystems in the southern and northern China, the two of most PAHs-polluted regions in China. *“Distribution and bioconcentration of polycyclic aromatic hydrocarbons in surface water and fishes”* studied the spatial distribution and bioconcentration of PAHs in the water, suspended solids and fish collected from Pearl River Delta in the southern China, while “*Distributions, sources, and backward trajectories of atmospheric polycyclic aromatic hydrocarbons at Lake Small Baiyangdian, northern China*” investigated the distributions, sources and backward trajectories of PAHs at Lake Small Baiyangdian in the northern China. The levels, distribution and health risks of PAHs in four edible fish species from the freshwater in the northern China were presented in *“Levels, distribution, and health risks of polycyclic aromatic hydrocarbons in four freshwater edible fish species from the Beijing market”*. The findings are of great importance to understand the transport and fate of PAHs in aqueous systems, and the associated health risks through fish consumptions. 

*“Fate and transport of polycyclic aromatic hydrocarbons in upland Irish headwater lake catchments”* presented the very important information on the fate and transport of PAHs in Upland Irish Headwater Lake catchments, since a few studies have evaluated the levels of PAHs within the Irish environment. Ireland, located on the western periphery of Europe and assumed to receive clean Atlantic air, has been used as an atmospheric reference for comparison to other Europe regions. 

In view of the importance of catchments to contribute pollutants to aquatic ecosystems, two papers (*“Fate and transport of polycyclic aromatic hydrocarbons in upland Irish headwater lake catchments”* and *“HCHs and DDTs in soils around Guanting Reservoir in Beijing, China: spatial-temporal variation and countermeasures”*) dealing with the PAHs and OCPs in the lake/reservoir catchment were selected to be published. 

The fresh water polluted by POPs would influence the seawater quality in coastal area. The sediments in the coastal area would record such effects. In view of this consideration, *“Concentration levels and ecological risks of persistent organic pollutants in the surface sediments of Tianjin coastal area, China”* that invested the concentration levels and ecological risks of PAHs, OCPs, and PBDEs in the surface sediments of Tianjin coastal area in the northern China was selected to be published in the POPFWE.

*Fu-Liu Xu* *Fu-Liu Xu* *Sven Erik Jorgensen* *Sven Erik Jorgensen* *Yoshihisa Shimizu* *Yoshihisa Shimizu* *Eugen Silow* *Eugen Silow*

